# The Influence of Nitrogen Flow on the Stoichiometric Composition, Structure, Mechanical, and Microtribological Properties of TiN Coatings

**DOI:** 10.3390/ma17010120

**Published:** 2023-12-26

**Authors:** Vasilina Lapitskaya, Andrey Nikolaev, Anastasiya Khabarava, Evgeniy Sadyrin, Pavel Antipov, Kamaludin Abdulvakhidov, Sergei Aizikovich, Sergei Chizhik

**Affiliations:** 1 Nanoprocesses and Technology Laboratory, A.V. Luikov Heat and Mass Transfer Institute, National Academy of Science of Belarus, 15 P. Brovki str., 220072 Minsk, Belarus; av.khabarova@mail.ru (A.K.); chizhik_sa@tut.by (S.C.); 2 Research and Education Center “Materials”, Don State Technical University, 1 Gagarin sq., 344003 Rostov-on-Don, Russia; andreynicolaev@eurosites.ru (A.N.); e.sadyrin@sci.donstu.ru (E.S.); sly_fox_03@mail.ru (P.A.); saizikovich@gmail.com (S.A.); 3 International Research Institute for Smart Materials, Southern Federal University, Sladkova 178/24, 344090 Rostov-on-Don, Russia; kgabdulvahidov@sfedu.ru

**Keywords:** TiN, coating, magnetron sputtering, stoichiometric composition, microstructure, mechanical properties, microtribological properties, coefficient of friction, specific volumetric wear, nanoindentation

## Abstract

Utilizing reactive DC magnetron sputtering method, TiN coatings were deposited on the silicon substrates at different nitrogen flows and powers. A study of the X-ray phase composition of the coatings was carried out. The stoichiometric composition of the coatings was determined using energy dispersive x-ray spectroscopy. The structure of the surface, cross-section, and thickness of the coatings were determined using scanning electron (SEM) and atomic force microscopy (AFM). A significant change in the surface structure of TiN coatings was established with changes in deposition power and nitrogen flow. SEM images of cross-sections of all coated samples showed that the formation of coatings occurs in the form of a columnar structure with a perpendicular orientation relative to the silicon substrate. The mechanical properties (elastic modulus *E* and microhardness *H*) of TiN coatings of the first group demonstrate a maximum at a nitrogen flow of 3 sccm and are 184 ± 11 GPa and 15.7 ± 1.3 GPa, respectively. In the second group, the values of *E* and *H* increase due to a decrease in the size of the structural elements of the coating (grains and crystallites). In the third group, *E* and *H* decrease. Microtribological tests were carried out in 4 stages: at a constant load, multi-cycle for 10 and 100 cycles, and with increasing load. The coefficient of friction (CoF) and specific volumetric wear ω depend on the roughness, topology, and mechanical properties of the resulting coatings. Fracture toughness was determined using nanoscratch and depends on the mechanical properties of TiN coatings. Within each group, coatings with the best mechanical and microtribological properties were described: in the first group—TiN coating at 3 sccm (with (29.6 ± 0.1) at.% N), in the second group—TiN coating at 2 sccm (with (40.8 ± 0.2) at.% N), and in the third group—TiN coating at 1 sccm (c (37.3 ± 0.2) at.% N).

## 1. Introduction

The development of multifunctional microelectromechanical systems (MEMS) requires longer service life of components and the device as a whole. Mechanical damage and rapid wear of contacting and rubbing surfaces can lead to disruption of the integrity and performance of MEMS elements and components and ultimately to failure of the entire device. The MEMS industry implies miniaturization, i.e., coatings of a few micrometers are not suitable for use in such devices [1]. Therefore, a crucial task of obtaining thin multifunctional coatings for microelectronics applications emerges.

Titanium nitride (TiN) coatings offer excellent mechanical, optical, and electrical properties [2,3], including bioinert and corrosion-resistant [4] ones. Thus, TiN coatings are widely used in medical applications [5,6] as well as aircraft and shipbuilding [7] to increase the wear resistance of cutting and processing tools [8,9]. For such applications, a large number of works on the characterization of TiN coatings deposited by various methods was fulfilled [10,11,12,13,14]. And to study the structure and properties, standard macromethods and equipment were used [9,15,16,17].

However, the use of TiN coatings is not limited to the areas mentioned above. The combination of several properties simultaneously opens up prospects for the usage of such coatings in microelectronics [18]. Thus, thin TiN coatings are already used in electrochemical microcapacitors (microsupercapacitors) as high-performance electrodes [19,20], and as anode material for thin-film Li-ion batteries [21]. However, the influence of the method and parameters of deposition of such thin coatings on their stoichiometric composition, structure, thickness, and properties remains important [1,16,22,23,24,25] to achieve the required parameters for use in certain devices. Adapting MEMS coatings requires studying the behavior and changing surface properties at the micro- and nanoscales [1]. The use of nanoindentation and nanoscratch testing to determine the properties of TiN coatings is a promising direction for studying mechanical and microtribological properties [1,10,13,26,27]. For example, using nanoindentation and mathematical modeling, the influence of crystallographic orientation on the microhardness of coatings was evaluated [10]. In [27], the wear-resistant properties of homogeneous (HM) and functionally graded (FG) TiN thin films were studied using nanoscratch testing. Utilizing nanoindentation, the deformation mechanisms and fracture behavior of TiN coatings were characterized [12]. In [13], nanoindentation and nanoscratch test methods were used to assess the influence of different nitrogen flow rates on the mechanical and microtribological properties of TiCrN coatings.

The purpose of the present work is to study the influence of nitrogen flow and deposition power in the chamber on the stoichiometric composition, microstructure, mechanical (including fracture toughness), microtribological, and wear-resistant properties of TiN coatings on a silicon substrate using high-precision probe methods.

## 2. Materials and Methods

TiN coatings were obtained by reactive DC magnetron sputtering of Ti (with a purity of 99.995%) using the VSM 100 (ROBVAC, Fryazino, Russia) magnetron system on silicon substrates of (100) orientation at a constant pressure of 0.78 Pa in the chamber of the system. The samples were sputtered in the spray power stabilization mode, which was 200, 300, and 465 W at a temperature of 200 °C (Table 1). The nitrogen flow varied for the first group from 1 to 15 sccm, for the second group—1, 1.5, 2 sccm, and for the third group—1 and 2 sccm (Table 1). Sputtering was carried out at a constant rotation speed of the samples. Before each sputtering, the target was cleaned by sputtering in an inert Ar atmosphere for 15 min at a sputtering power of 100 W.

After deposition, the coated samples were cleaved along a previously applied notch on the back side of the Si substrate. The thickness of the coatings was further determined using a Crossbeam 340 scanning electron microscope (SEM, Carl Zeiss Microscopy, Oberkochen, Germany). During the study, the samples were fixed in a special holder so that the surface of the cross-section was located normally under the electron beam. The studies were carried out using an Everhart-Thornley secondary electron detector with an extra high tension (EHT) of 3 kV. After this, the samples were glued to the positioning table using conductive tape and the microstructure of their surface was studied; the EHT was 3 kV as well.

The phase analysis was performed on an X-ray diffractometer (D2 Phaser, Bruker, Karlsruhe, Germany) using Cu Ka (Kα_1_ = 1.54060 Å, Kα_2_ = 1.54443 Å, Kα_2_/Kα_1_ ratio = 0.50) radiation with a step size of Δ2θ = 0.01° and a data acquisition time of τ = 1 s. The voltage on the X-ray tube was set to 30 kV, and the current was set to 10 mA.

Microstrains were determined from the relative full width at half-maximum broadening of the most intense reflection at high angles of 2θ XRD spectra, corresponding to the predominant TiN phase in the coating.

The microstructure and roughness of the coatings was assessed using a Dimension FastScan (Bruker, Santa Barbara, CA, Billerica, MA, USA) atomic force microscope (AFM) in the PeakForce QNM (Quantitative Nanoscale Mechanical Mapping) mode. The standard CSG10_SS silicon cantilevers (TipsNano, Moscow, Russia) with a cantilever stiffness of 0.3 and 1.1 N/m and a tip radius of 3 and 5 nm were used.

The stoichiometric composition and chemical analysis of the coatings were assessed using an EDX Oxford X-Max 80 (Oxford Instruments, Abingdon, UK) with 200× magnification and a voltage of 20 kV from the sample surface over the entire area with a wide coverage (approximately ~150–200 µm by ~400–500 µm), as well as from individual contrast crystals pointwise.

The mechanical properties were determined using a Hysitron 750Ubi (Bruker, Minneapolis, MN, USA) nanoindentation system. A diamond conical indenter with a radius of curvature of 226 nm and an angle of 60° at the apex was used. The mechanical properties were determined at a constant load of 1 mN (the results were averaged for 9 indentations for each sample). The indentation depth did not exceed 1/10 of the coating thickness.

The tribological properties were determined using the above-described Hysitron 750Ubi nanoindentation system with a diamond conical indenter (R = 226 nm). To conduct tribological tests (nanoscratch testing), the nanoindenter was equipped with a two-dimensional transducer. Tests were carried out using specified functions of load and scratch length. During testing, 3 scratches 6 µm long were applied to each sample in 15 sec with a constant load of 0.5, 0.75, and 1.0 µN for TiN coatings. Multi-cycle tests consisted of applying 3 scratches of 6 μm length in 10 cycles of 5 s/cycle (total path 60 μm, duration 50 s) at a load of 1 mN for TiN coatings. The process of applying 3 scratches with a length of 5 μm in 100 cycles of 5 s/cycle (total path 500 μm, duration 500 s) was carried out with a load of 100 μN for all samples. Scratching of the surface with an increasing load up to 5000 μN (5 mN) was carried out for TiN coatings over a distance of 5 μm in 5 s. The specific volumetric wear (ω) after 100 cycles was calculated from the volume of material removed during the friction test divided by the product of load and distance. The temperature and relative humidity in the nanoindentation and nanoscratch tests were 21 ± 2 °C and 45%, respectively.

Based on nanoscratching using a nanoindenter at a constant load of 1 mN, the fracture toughness *K*_IC_ of TiN coatings was calculated. For this purpose, the following formula was used:

(1) $$K_{\mathrm{IC}}=\frac{F_{eq}}{\sqrt{2dw+4d^{2}w}}$$

where *F_eq_*—driving force of fracture, H; *d*—depth of the track, m; *w*—width of the track, m [28]. Driving force of fracture *F_eq_* is calculated as in [28]:

(2) $$F_{eq}=\sqrt{F_{T}^{2}+\frac{3}{5}F_{V}^{2}}$$

where *F_T_*—vertical (normal) load and *F_V_*—horizontal (lateral) force (N), held during the nanoscratching process [28].

## 3. Results and Discussion

**Figure 1:**
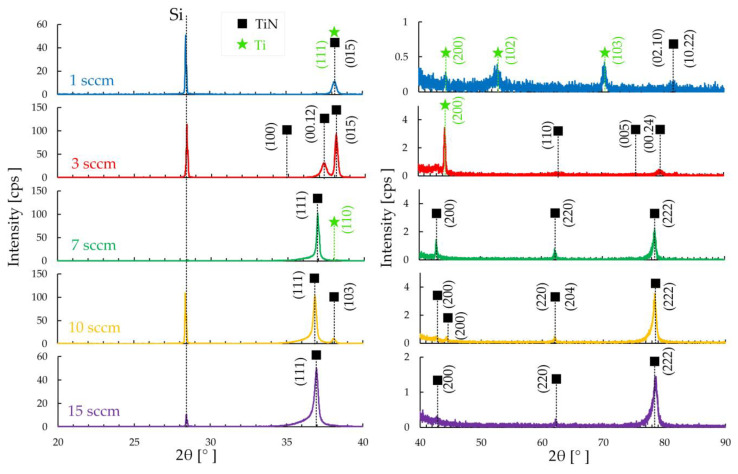
XRD for the TiN coatings of group 1.

In the diffraction patterns of the deposited TiN coatings of the first group, diffraction lines are observed originating from the trigonal, tetragonal, and cubic phases of TiN (Figure 1). Thus, the coating deposited at 1 sccm is represented by trigonal TiN, at 3 and 10 sccm—tetragonal TiN, and at 7 and 15 sccm—cubic TiN (Figure 1, Table 2). In the coating deposited at 1 sccm, the Ti (111) phase predominates. With an increase to 3 sccm, TiN (015) becomes the predominant phase, and the Ti (200) phase remains in the composition. The coating also contains TiN phases (100), (00.12), (110), (005), and (00.24). A further increase in nitrogen to 7–15 sccm leads to the formation of TiN phases (111), (112), (200), (220), (222), and (224) [29]. It should be noted that only the coating deposited at 3 sccm contains TiN phases (00.12) and (00.24). At a nitrogen flow of 7 to 15 sccm, a peak (222) appears in the composition and its intensity increases with increasing flow.

**Figure 2:**
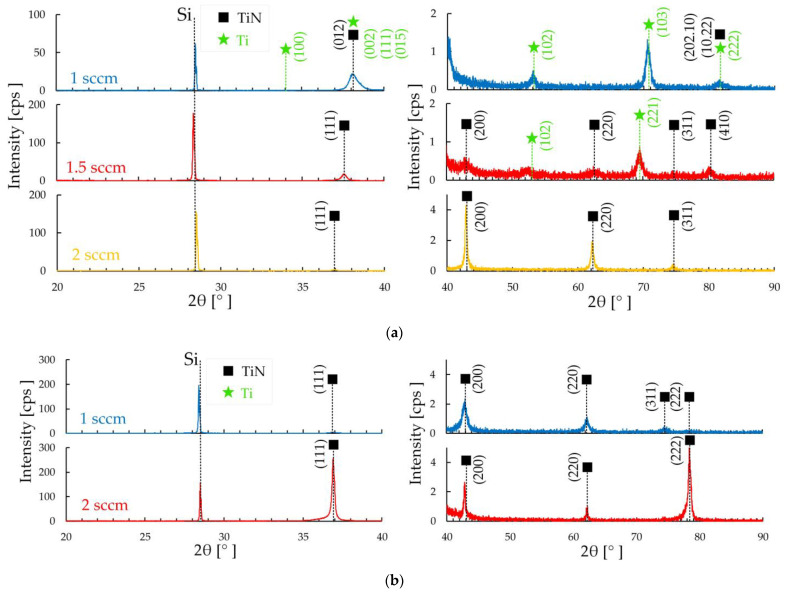
XRD for the TiN coatings of groups 2 (**a**) and 3 (**b**).

Diffraction patterns of groups 2 and 3 for the most part show the presence of the TiN cubic phase (Figure 2). Only in the coating of group 2, deposited at 1 sccm, the TiN phase is presented in a trigonal form (Figure 2a).

The cubic phase is represented by TiN with orientation (111), (200), (220), and (311). The predominant cubic phase on the coating of group 2 deposited at 1.5 sccm is the (111) orientation. With an increase to 2 sccm, the peak of this orientation decreases (Figure 2a). Peak (200) also increases with flux changing from 1.5 to 2 sccm. On the coatings of group 3, the intensity of the (111) peak increases with increasing nitrogen flow from 1 to 2 sccm (Figure 2b). Peak (222) behaves similarly—it increases with increasing nitrogen flow from 1 to 2 sccm.

Based on the X-ray phase analysis results, the presence of insignificant microstresses Δ*d*/*d* in each coating was determined (Table 2). For the coatings deposited at 1 sccm in each group, they have Δ*d*/*d* = 2.5 × 10^−4^, and with increasing flow in all groups, the ratio increases to Δ*d*/*d =* 2.6 × 10^−4^.

According to the results of EDX of the TiN coatings, the atomic content of nitrogen in the coating composition increases and titanium content decreases (Table 3).

In the first group of coatings, when the nitrogen flow changes from 1 to 7 sccm, the nitrogen content changes from 8.6 ± 0.2 at. % N up to 44.6 ± 0.2 at. % N. A further increase in flow practically does not lead to a change in the nitrogen content in the coating composition. A similar slight change in nitrogen content at a nitrogen flow of 7 sccm and higher was noted in [18]. In the second group of coatings, the content changes from 2.6 ± 0.3 at. % N at 1 sccm to 40.8 ± 0.2 at 2 sccm (Table 3).

**Figure 3:**
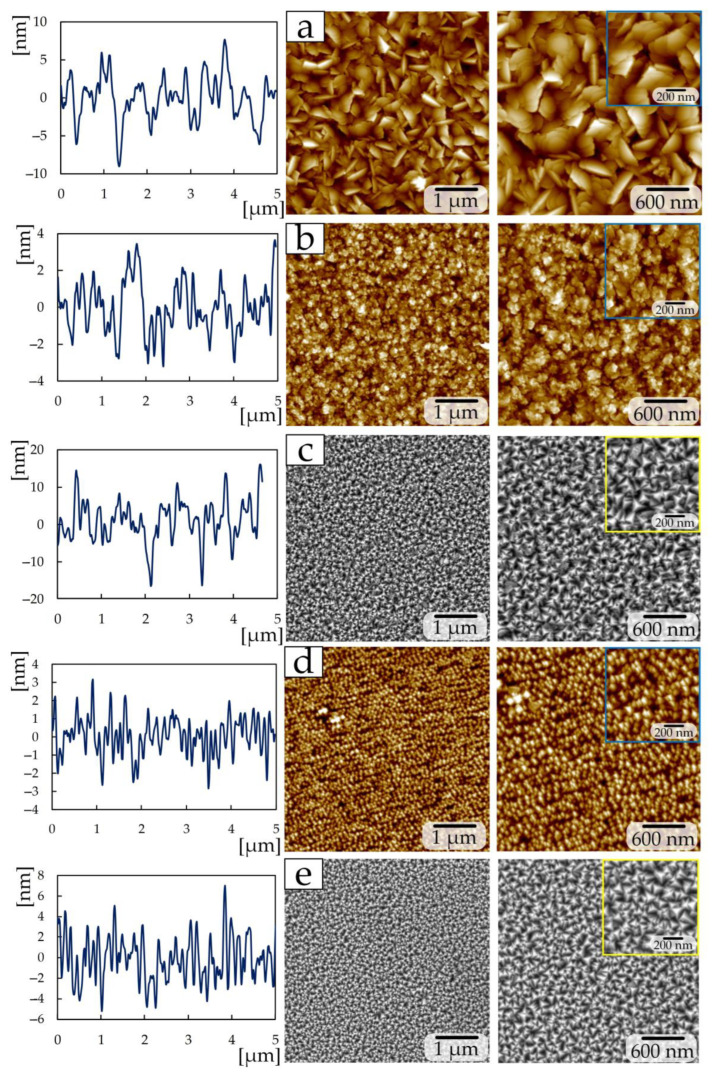
AFM (**a**,**b**,**d**) and SEM (**c**,**e**) images for the TiN coatings of group 1: (**a**) 1 sccm; (**b**) 3 sccm; (**c**) 7 sccm; (**d**) 10 sccm; (**e**) 15 sccm.

**Figure 4:**
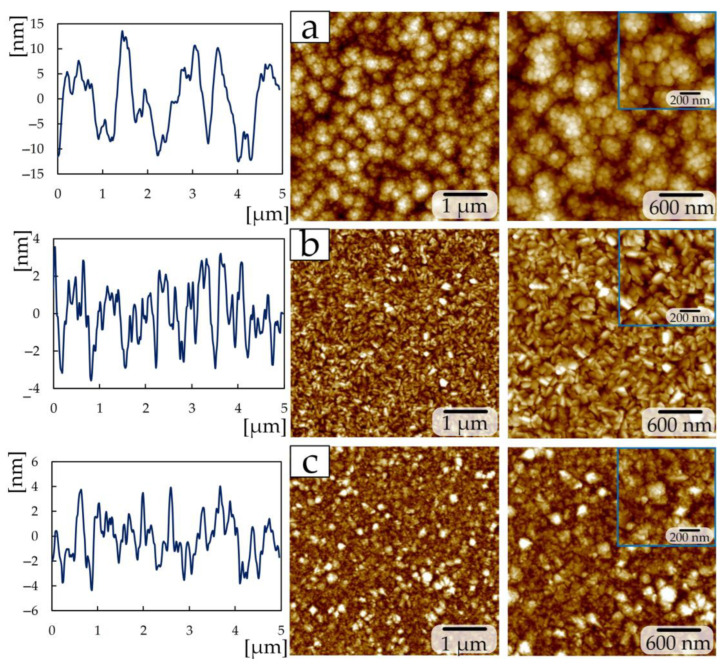
AFM images for the TiN coatings of group 2: (**a**) 1 sccm; (**b**) 1.5 sccm; (**c**) 2 sccm.

**Figure 5:**
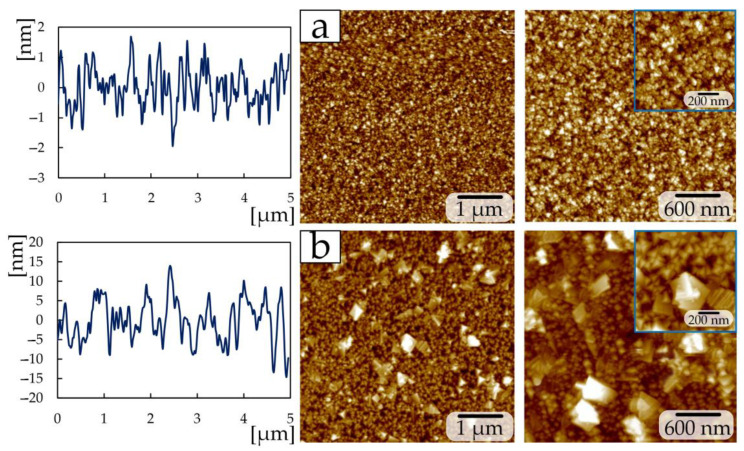
AFM images for the TiN coatings of group 3: (**a**) 1 sccm; (**b**) 2 sccm.

The surface structure of samples with TiN coatings differs significantly with changes in deposition power and nitrogen flow (Figure 3, Figure 4 and Figure 5). The surface of the TiN coating of the first group with 1 sccm consists of plates with sharp edges (Figure 3a). Such a structure is similar to the structure of a TiN film deposited by the magnetron sputtering method under nitrogen pressure 2 × 10^−4^ mbar [16].

Increasing the nitrogen flow to 3 sccm leads to the formation of a “flaky” surface structure (Figure 3b), and a further increase in the flow to 7–15 sccm makes it possible to obtain triangular-shaped crystallites on the surface (Figure 3c–e). In this case, the crystallite size decreases with increasing flow from 100 to 135 nm to 82 to 21 nm. The observed decrease in crystallite size is associated with a decrease in the intensity of the (111) peak of TiN (Figure 1), similar to the research [30]. This structure is similar to the structure of TiN coatings deposited by the magnetron method in [14,15]. At the same time, in [14], during deposition, the power changed from 80 to 200 at a constant nitrogen flow of 2 sccm, and the change in power affected the change in crystallite sizes [14]. And in [15], the nitrogen flow was 17 sccm.

Reducing the power from 465 to 300 W during the deposition of TiN coatings of the second group made it possible to obtain a structure (Figure 4) that differs from group 1. The sample at a nitrogen flow of 1 sccm has a granular structure (Figure 4a), with grains of 50–100 nm in size collected in large conglomerates measuring 300–600 nm. A similar grain structure was obtained in [19] by the magnetron sputtering method at a nitrogen pressure of 3.5 sccm. Increasing the nitrogen flow to 1.5 sccm makes it possible to obtain a plate-like structure on the surface, the edges of which have a rectangular shape (Figure 4b). The nitrogen flow in the 2 sccm chamber produces a fine-grained structure (grain size 20–70 nm) which included diamond-shaped crystallites up to 300 nm in size (Figure 4c).

Samples deposited at a power of 200 W and a nitrogen flow of 1 sccm (Figure 5a) have a similar structure (granular with a minor presence of crystallites 60–90 nm in size) to a sample from group 2 at 2 sccm (Figure 4c), and a sample deposited at 2 sccm (Figure 5b) consists of triangular-shaped crystallites as in the samples of the first group at 7–15 sccm (Figure 3c–e). The size of triangular crystallites on sample 2 sccm of the third group is 80–100 nm (Figure 5b). In addition to triangular crystallites, the surface of sample 2 sccm of the third group contains structures up to 500 nm in size, consisting of multiple plates.

The surface roughness Ra (Table 3) of the first group of samples decreases from 16.4 ± 0.8 nm to 2.4 ± 0.1 nm with an increase in nitrogen flow from 1 to 15 sccm, respectively. Changing the nitrogen flow by 1 sccm and reducing the power to 300 W in the second group of coatings leads to a decrease in roughness by almost 2.5 times. The high roughness on a sample with 1 sccm of the second group compared to samples of 1.5 and 2 sccm depends on the size of the conglomerates. In this group of samples, the increase in crystallite size is associated with an increase in the intensity of the (111) TiN peak [30] according to XRD (Figure 2b).

A further reduction in power to 200 W for the third group at the same nitrogen flows (1 and 2 sccm) increases the roughness by 3.2 times. The increase in roughness is associated with the presence of plate-like structures on the surface up to 500 nm in size (Figure 5b).

**Figure 6:**
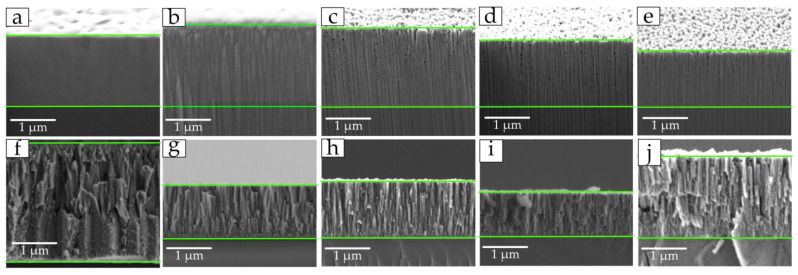
SEM images of cross-sections of TiN coatings of group 1 (**a**–**e**), group 2 (**f**–**h**), and group 3 (**i**,**j**): (**a**) 1 sccm; (**b**) 3 sccm; (**c**) 7 sccm; (**d**) 10 sccm; (**e**) 15 sccm; (**f**) 1 sccm; (**g**) 1.5 sccm; (**h**) 2 sccm; (**i**) 1 sccm; (**j**) 1.5 sccm.

SEM images of cross-sections of all coated samples showed that the formation of coatings occurs in the form of a columnar structure with a perpendicular orientation relative to the silicon substrate (Figure 4). Samples with triangular crystallites on the surface have a more uniform and ordered column structure in the cross-section (Figure 6c–e).

On all other samples, the uniformity and width of the columns is chaotic. The samples, as in [14,16], are characterized by a non-equiaxial columnar structure. It should be noted that in the cross-section of samples of the second group, the width of the columns decreases with increasing nitrogen flow (Figure 6f–h).

**Figure 7:**
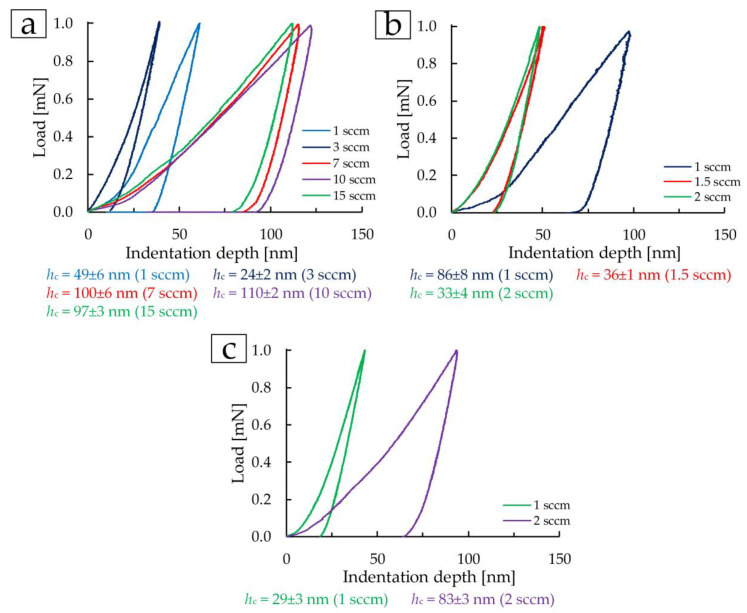
Indentation curves at 1 mN load for the TiN coatings of group 1 (**a**), group 2 (**b**), and group 3 (**c**).

**Figure 8:**
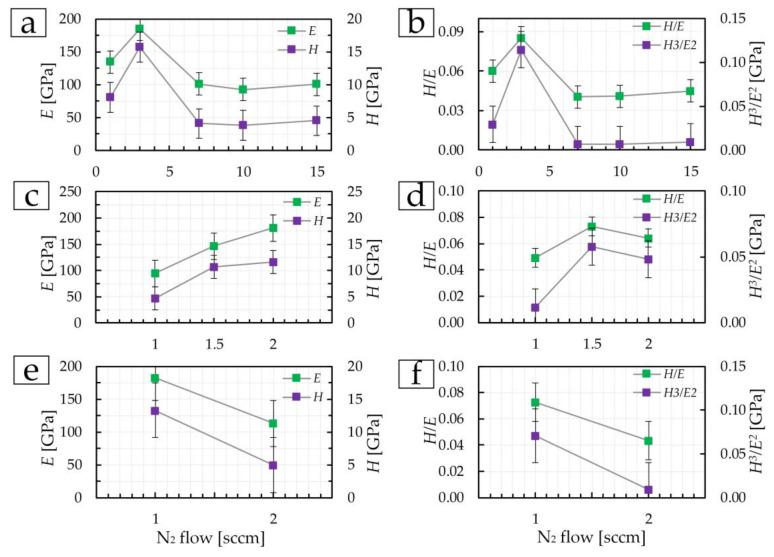
The mechanical properties of TiN coatings of group 1 (**a**,**b**), group 2 (**c**,**d**), and group 3 (**e**,**f**).

Mechanical properties measured by nanoindentation at a load of 1 mN are demonstrated in Figure 7 and Figure 8. The indentation diagrams show that in the first group, coatings deposited at 7, 10, and 15 sccm demonstrate similar mechanical properties (Figure 7a). The coating deposited at 3 sccm shows the most elastic properties. In groups 2 and 3, the most elastic coatings were deposited at 2 and 1 sccm, respectively (Figure 7b,c). Also, on these coatings, the smallest indentation depth was obtained at a load of 1 mN (Figure 7).

The mechanical properties, namely, elastic modulus *E* and microhardness *H*, of the TiN coatings for the first group at 1 sccm are 135 ± 9 GPa and 8.1 ± 0.9 GPa, respectively (Figure 8a). An increase in nitrogen flow from 1 to 3 sccm increases *E* and *H* to 184 ± 11 GPa and 15.7 ± 1.3 GPa, respectively. With an increase in nitrogen flow in the range of 7–15 sccm, the mechanical properties decrease and practically do not change. At 15 sccm, *E* and *H* are 101 ± 4 GPa and 4.5 ± 0.1 GPa, respectively. In the range from 20 to 30 at.% N (corresponding nitrogen flow from 3 to 6 sccm) in the coating composition in [18], a maximum of *E* and *H* values is noted. A similar change occurs in the first group of coatings in this work. The sharp difference in the physical and mechanical properties of the coating deposited at 3 sccm can be explained by a different composition according to XRD and the presence of (00.12), (100) TiN phases.

In group 2, the elastic modulus and microhardness increase with increasing nitrogen flow (Figure 8c). At 1 sccm, the elastic modulus was 94 ± 8 GPa, and the microhardness was 4.7 ± 0.4 GPa. At a flow of 2 sccm, *E* and *H* increased by 2 and 2.5 times, respectively. This is due to a decrease in the structural elements of the coating—grains and crystallites (Figure 4a,c).

The coating deposited at a flow of 1 sccm and 200 W (third group) is characterized by *E* and *H* values (Figure 8e) close to the values of the TiN coating deposited at 3 sccm and 465 W (first group) and are 183 ± 8 GPa and 13.3 ± 1.4 GPa. Both the structure (Figure 3b) and the mechanical properties of the TiN coating (Figure 8e) at a nitrogen flow of 2 sccm in the third group of coatings are almost similar to the samples obtained at 7–15 sccm in the first group of coatings (Figure 8a) and amount to *E* = 112 ± 4 GPa and *H* = 4.9 ± 0.2 GPa. Additionally, let us denote that a decrease in physical and mechanical properties in this group of coatings is associated with an increase in the size of crystallites and grains due to an increase in the intensity of the (111) peak, i.e., the Hall–Petch effect (as the grain size increases, the hardness of the material decreases).

The values of the elastic modulus of the coatings in this work are close to the values of TiN coatings deposited by the magnetron method with varying deposition time and temperature in [13], in which the values vary in the range from 87 to 136 GPa. The microhardness in [13] in the range from 8.7 to 19.3 GPa. Also, similar values were obtained in [31], in which *H* and *E* increased with increasing deposition time and coating thickness.

The coefficients *H*/*E* (the resistance to elastic deformation (plasticity index)) and *H*^3^/*E*^2^ (the resistance of the material to plastic deformation) change similarly (Figure 8b,d,f) to the modulus of elasticity and microhardness of coatings.

The microtribological properties of the coating surfaces depend both on the roughness parameters and topology, as well as the mechanical properties. Microtribological tests were carried out in several stages.

**Figure 9:**
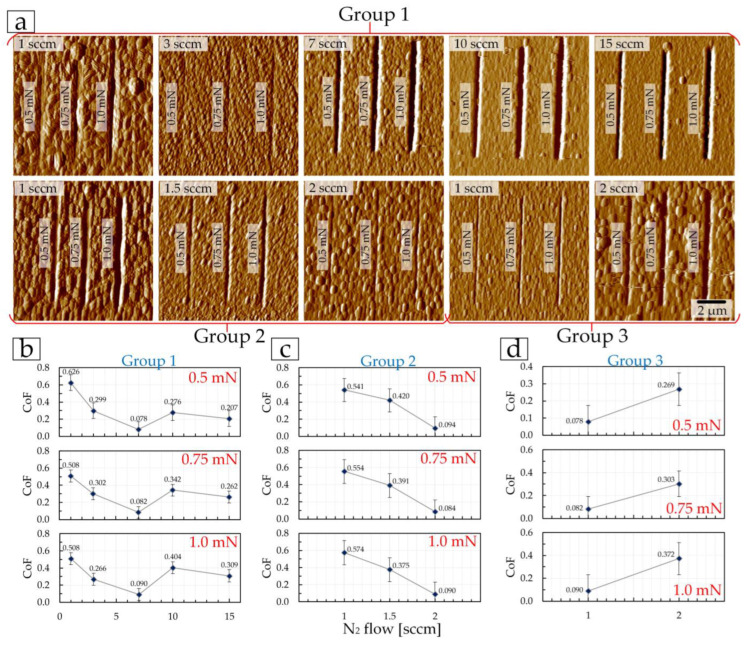
AFM images ((**a**), 10 × 10 μm^2^) of the surface of TiN coatings after a nanoscratch test at constant loads (0.5, 0.75, and 1.0 mN) and the dependence of the friction coefficient on the nitrogen flow (**b**–**d**).

The first stage included conducting a nanoscratch test at a constant load of 0.5, 0.75, and 1 mN. In this case, each load corresponded to one scratch on the surface, i.e., each coating received three scratches under three loads (Figure 9a).

At each load, the trend in the coefficient of friction as a function of nitrogen flow is maintained within each coating group (Figure 9b–d). In the first group of coatings, CoF decreases from 0.626 (at 0.5 mN) and 0.508 (at 0.75 and 1.0 mN) to 0.078 (at 0.5 mN) and 0.090 (at 1.0 mN) when the nitrogen flow changes from 1 to 7 sccm (Figure 9b). In this case, friction CoF correlates with surface roughness (Table 3). Next, the correlation of CoF with roughness changes to a correlation with the mechanical properties. On the coating at 10 sccm of the first CoF group, friction increases (*E* and *H* decrease) to 0.276 (at 0.5 mN) and 0.404 (at 1.0 mN), and at 15 sccm—CoF decreases slightly (with a slight increase in *E* and *H*) (Figure 9b).

In the second group of CoF coatings, friction decreases at all loads from 0.541 (at 0.5 mN) and 0.574 (at 1.0 mN) to 0.084 (at 0.75 mN) and 0.094 (at 0.5 mN) when the nitrogen flow increases from 1 to 2 sccm. In this group of coatings, there is a correlation with both roughness (Ra and Rq decrease, Table 3) and mechanical properties (*E* and *H* increase, Figure 9c). A similar correlation of CoF with the mechanical properties and surface roughness is observed for the third group of TiN coatings (Figure 9d). As the nitrogen flow increases, the friction CoF increases. At the same time, the roughness also increases (Table 3), and *E* and *H* decrease (Figure 8e). It should be noted that the depth of scratches (Figure 9a) is the smallest for coatings with maximum values of *E* and *H* within their groups: in the first group—TiN coating at 3 sccm, in the second group—TiN coating at 2 sccm, and in the third group—TiN coating at 1 sccm.

**Figure 10:**
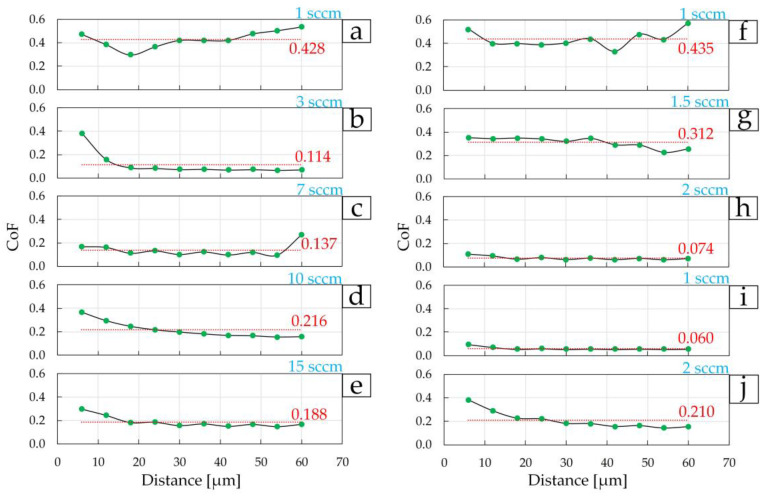
Change in the coefficient of friction during the first 10 cycles on TiN coatings of group 1 (**a**–**e**), group 2 (**f**–**h**), and group 3 (**i**,**j**).

**Figure 11:**
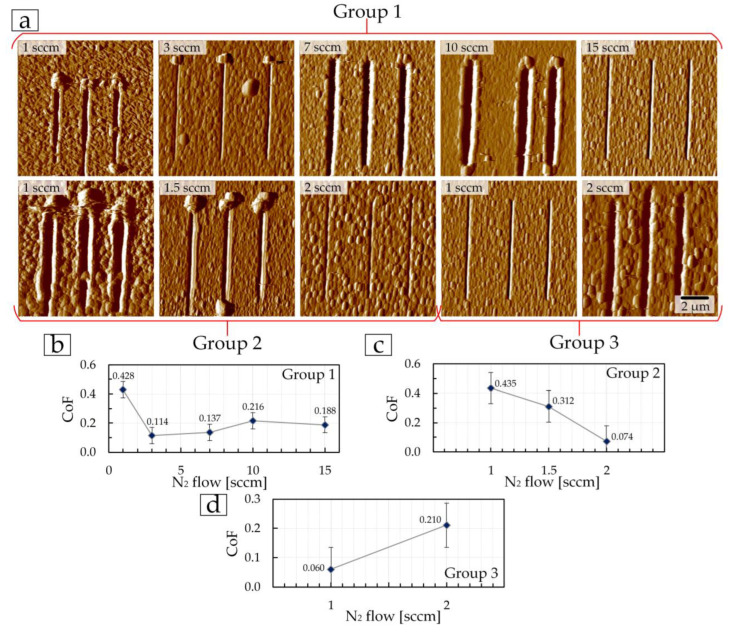
AFM images ((**a**), 10 × 10 µm^2^) of the surface of TiN coatings after a multi-cycle (10 cycles) nanoscratch test and the dependence of the resulting average friction coefficient on the nitrogen flow (**b**–**d**).

The second stage of testing consisted of studying the change in the coefficient of friction CoF during high-cycle friction over the first 10 cycles, when the counter-body (in our case, a diamond conical indenter) is significantly influenced by the topology and all surface irregularities. Three scratches were performed on each coating for 10 cycles (Figure 10 and Figure 11). The average value for each coating was then determined.

Figure 10 shows the dependence of CoF on the distance that the diamond indenter travels during 10 test cycles (60 μm). The average CoF value is plotted in red on each diagram (Figure 10). In such a manner, one can study how CoF changes relative to the average value, i.e., shows the “resistance” of the coating to nanoscratching. The most stable CoF are those coatings that demonstrated high values of *E* and *H*, as well as low CoF at the first stage of tribological testing. In the first group of coatings, the lowest average CoF = 0.114 value was obtained on the coating deposited at 3 sccm (Figure 10b and Figure 11b). Relatively low fluctuations in values relative to the average values were also found for coatings at 7–15 sccm in the first group, which is associated with low surface roughness. The lowest CoFs of 0.074 and 0.060 were obtained on coatings deposited at 2 sccm of the second group (Figure 10i and Figure 11c) and 1 sccm of the third group (Figure 10j and Figure 11d), respectively. Figure 11a shows images of three nanoscratches on each coating after a 10-cycle test, as well as the dependence of the average CoF values on changes in nitrogen flow during deposition of the coatings. Similarly, in the first stage, the smallest depth of nanoscratches is observed on the TiN coating in the first group, deposited at 3 sccm, in the second group—TiN coating at 2 sccm, and in the third group—TiN coating at 1 sccm.

**Figure 12:**
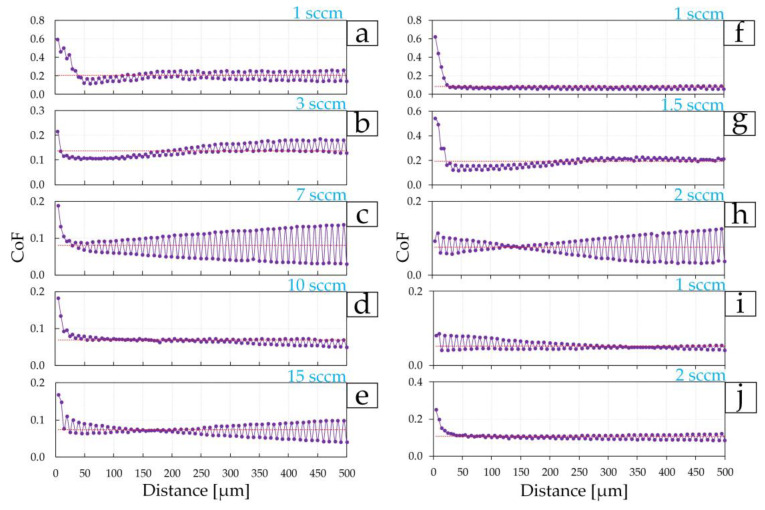
Change in the coefficient of friction at 100 cycles on TiN coatings of group 1 (**a**–**e**), group 2 (**f**–**h**), and group 3 (**i**,**j**).

**Figure 13:**
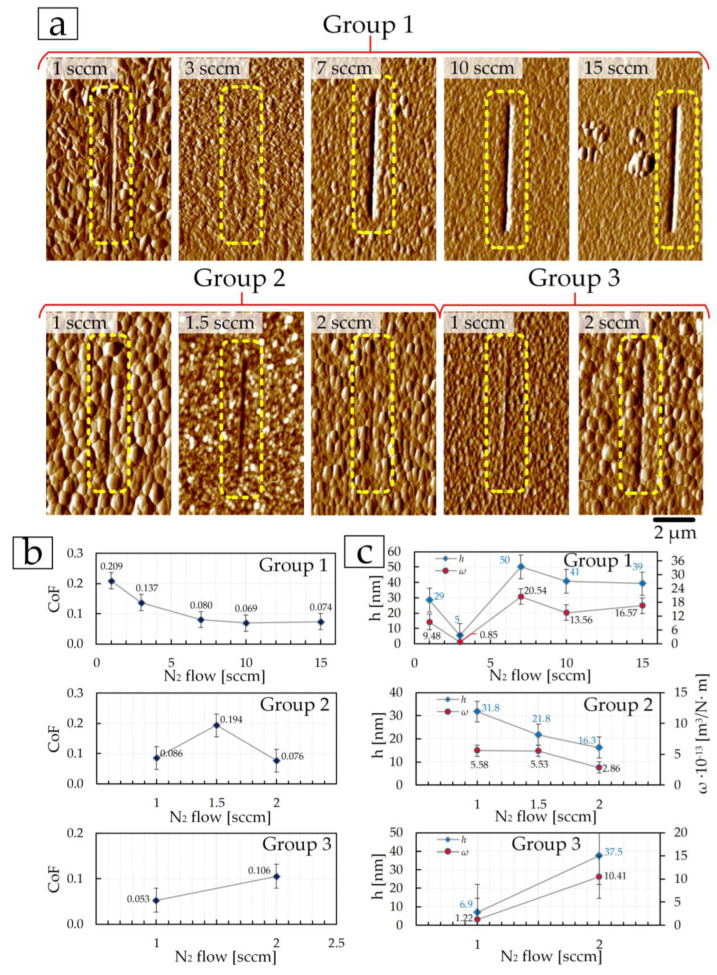
AFM images ((**a**), 10 × 10 µm^2^) of the surface of TiN coatings after a multi-cycle (100 cycles) nanoscratch test, the dependence of the resulting average friction coefficient, depth, and specific volumetric wear on the nitrogen flow (**b**,**c**).

The third stage of tribological testing consisted of a multi-cycle test of 100 cycles with a total distance of 500 μm on each coating. High values of the first 60–70 µm are associated, as previously mentioned, with the significant influence of roughness and all surface irregularities, because at the micro level, the diamond indenter interacts with each asperity separately. Then the surface is smoothed with constant impact of the indenter on the surface and the CoF of friction becomes more stable until the coating begins to fail during tribological tests. However, such changes occur differently on each surface. For example, the coating of the first group, deposited at 3 sccm, which showed the best mechanical properties and low friction CoF at the first and second stages of tribological tests, did not show the lowest CoF value at 100 tribological test cycles. After 150 µm (30 cycles), the CoF changes relative to the average value of 0.137 on this coating increase (Figure 12b). The minimum fluctuations in CoF values in the first group of coatings were shown by the coating deposited at 10 sccm (Figure 12d). The largest fluctuation in CoF values was found for the coating of the first group, deposited at a nitrogen flow of 7 sccm. The change in average CoF values (after 100 cycles) with nitrogen flow for the first group (Figure 13b) of coatings directly correlates with surface roughness (Table 1).

In the second group, coatings deposited at 1 and 1.5 sccm have small changes in CoF values relative to the average values of 0.086 and 0.184, respectively (Figure 10b and Figure 12f,g) throughout the entire test distance. In this case, the lowest average CoF value of 0.076, but with a large fluctuation in CoF values relative to the average, was obtained on the coating deposited at 2 sccm (Figure 12i and Figure 13b).

Average CoF values (after 100 cycles) for coatings of the third group correlate with the mechanical properties and roughness—with an increase in nitrogen flow from 1 to 2 sccm, CoF increases (Figure 13b) due to an increase in surface roughness (Table 3), and a decrease in *E* and *H* (Figure 8e,f). A lower average CoF value of 0.053 after 100 cycles of tribological tests was obtained on the coating deposited at 1 sccm.

During this stage of testing, the depth *h*, and the width of the wear tracks were determined as well, and the specific volumetric wear ω was calculated. Based on the images of wear tracks after 100 cycles (Figure 13a), the depth of the tracks, and the obtained values of specific volumetric wear (Figure 13c), it was established that the least wear occurred on coatings deposited in the first group—at 3 sccm, in the second—at 2 sccm, in the third—at 1 sccm.

The resulting depth *h* for these coatings was, respectively, the following: 5 ± 1, 16 ± 5, and 7 ± 1 nm, and the specific volumetric wear *ω* was: (0.85 ± 0.04) × 10^−13^ m^3^/N·m, (2.86 ± 0.14) × 10^−13^ m^3^/N·m, and (1.22 ± 0.06) × 10^−13^ m^3^/N·m.

The influence of the mechanical properties on the tribological properties (reduction of CoF and specific volumetric wears) of TiN coatings was shown in [11,24], and the influence of topography and surface roughness was shown in [10].

**Figure 14:**
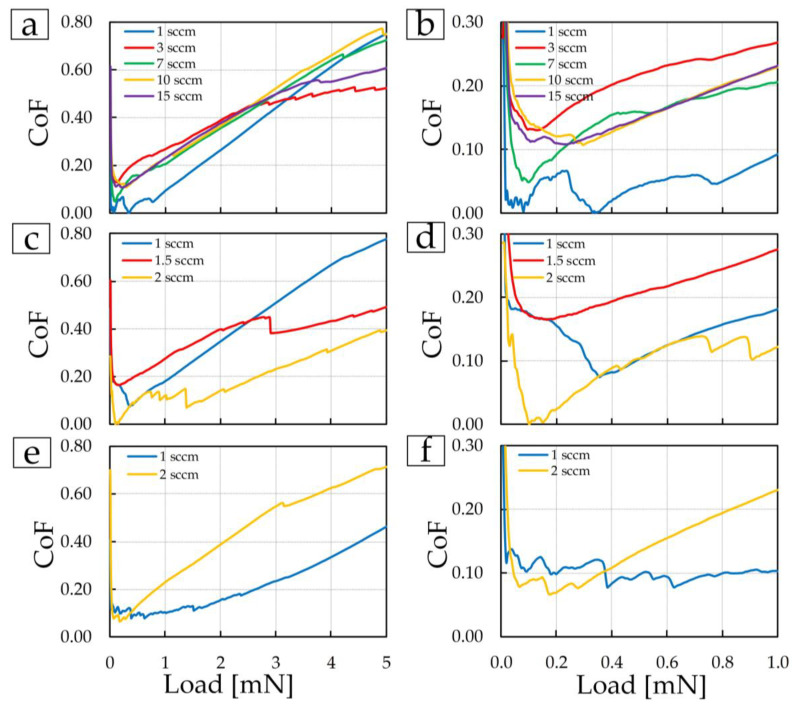
Dependence of CoF on load: (**a**,**c**,**e**) from 0 to 5 mN; (**b**,**d**,**f**) from 0 to 1 mN.

The fourth stage of tribological testing was to conduct a nanoscratch test with an increasing load from 0 to 5 mN per scratch 5 µm long in 5 s (Figure 14). Such tests make it possible to show the behavior of coatings under suddenly occurring critical loads.

As in all previous tests, coatings deposited at 3 sccm in the first group (red line in Figure 13a,b), 2 sccm in the second group (yellow line in Figure 13c,d), and 1 sccm in the third group (Figure 13e,f blue line) compared to other coatings showed the most stable properties and at maximum load have a lower CoF.

**Figure 15:**
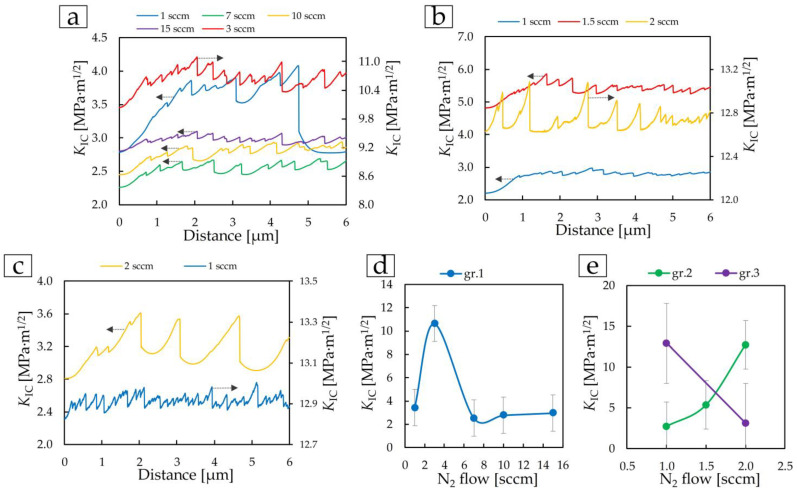
Dependence of fracture toughness *K*_IC_ (**a**–**c**) on distance and average *K*_IC_ values on TiN coatings of group 1 (**d**), group 2, and group 3 (**e**).

The fracture toughness *K*_IC_ was determined from the resulting scratches (Figure 9a) at a load of 1 mN. Dependences (Figure 15) of changes in *K*_IC_ on distance (scratch length 6 μm), as well as dependences of *K*_IC_ on the nitrogen flow of deposited coatings (Figure 15d) were obtained.

Fracture toughness fully correlates with the mechanical properties of coatings. The microtribological properties of the surface have less influence on the fracture toughness determined by the nanoscratch test. The TiN coating of the first group, deposited at 3 sccm, had the highest value of *K*_IC_ = 10.6 ± 1.2 MPa·m^1/2^, as well as mechanical properties. Almost identical *K*_IC_ values of 12.7 ± 1.4 MPa·m^1/2^ and 12.9 ± 1.4 MPa·m^1/2^ were obtained on coatings of groups 2 and 3 deposited at 2 and 1 sccm, respectively.

## 4. Conclusions

Using the reactive DC magnetron sputtering method, TiN coatings were deposited on silicon substrates at different nitrogen flows. Additionally, the power in the chamber during coating deposition changed—200, 300, and 465 W. As a result, three groups of coatings were obtained. Using EDX, the stoichiometric composition of the coatings was determined. The structure of the surface, cross-section, and thickness of the coatings was determined using scanning electron and atomic force microscopy.

A significant change in the surface structure of TiN coatings was established with changes in deposition power and nitrogen flow. According to X-ray phase analysis of the deposited TiN coatings of the first group, diffraction lines were observed originating from the trigonal, tetragonal, and cubic phases of TiN. The coating deposited at 3 sccm demonstrates a phase composition different from all others: the composition contains TiN phases (015), (200), (100), (00.12), (110), (005), and (00.24). A further increase in nitrogen to 7–15 sccm leads to the formation of common TiN phases (111), (112), (200), (220), (222), and (224), most of which are present in the coatings of groups 2 and 3. The structure of coatings of the first group changes from lamellar to crystallite, where each crystallite is triangular in shape. In the second group, the granular structure with conglomerates changes to a fine-grained structure which included diamond-shaped crystallites, and in the third group, the granular structure with included crystallites changes to triangular-shaped crystallites with inclusions from many plates. SEM images of cross-sections of all samples with coatings showed that the formation of coatings occurs in the form of a columnar structure with a perpendicular orientation relative to the silicon substrate. The change in the size of crystallites and grains is explained by a change in the intensity of the peak of the (111) TiN phase.

The mechanical properties (elastic modulus *E* and microhardness *H*) of TiN coatings of the first group have a maximum nitrogen flow of 3 sccm and are 184 ± 11 GPa and 15.7 ± 1.3 GPa, respectively. Such a phenomenon can be explained by a different phase composition. In the second group, the values of *E* and *H* increase due to a decrease in the size of the structural elements of the coating (grains and crystallites). In the third group, *E* and *H* decrease to 112 ± 4 GPa and 4.9 ± 0.2 GPa, respectively. Microtribological tests were carried out in 4 stages: at a constant load, multi-cycle for 10 and 100 cycles, and with increasing load. The friction coefficient and specific volumetric wear depend both on the roughness and topology and the mechanical properties of the resulting coatings. Within each group, coatings with the best mechanical and microtribological properties were identified: in the first group—TiN coating at 3 sccm (with (29.6 ± 0.1) at.% N), in the second group—TiN coating at 2 sccm (with (40.8 ± 0.2) at.% N), and in the third group—TiN coating at 1 sccm (with (37.3 ± 0.2) at.% N). The wear depth *h* for these coatings was as follows: 5 ± 1, 16 ± 5, and 7 ± 1 nm, and the specific volumetric wear ω was: (0.85 ± 0.04) 10^−13^ m^3^/N m, (2.86 ± 0.14)·10^−13^ m^3^/N·m, and (1.22 ± 0.06)·10^−13^ m^3^/N·m. It has been established that fracture toughness, determined by nanoscratch testing, depends on mechanical properties.

## Figures and Tables

**Table 1:** TiN coating deposition parameters.

Group	Sample No.	N_2_ Flow (sccm)	Ar Flow (sccm)	Power (W)	Temperature (°C)	Deposition Time (min)
1	1.1	1	84	465	200	40
	1.2	3	82			
	1.3	7	78			
	1.4	10	75			
	1.5	15	70			
2	2.1	1	85			
	2.2	1.5		300		
	2.3	2				
3	3.1	1	80	200		
	3.2	2				

**Table 2:** Phases in coatings (according to XRD).

Group	N_2_ Flow (sccm)	Phase	Δ*d*/*d* (Microstrains)
1	1 sccm	Trigonal TiN	2.5 × 10^−4^
	3 sccm	Tetragonal TiN	2.5 × 10^−4^
	7 sccm	Cubic TiN	2.6 × 10^−4^
	10 sccm	Tetragonal TiN	2.6 × 10^−4^
	15 sccm	Cubic TiN	2.6 × 10^−4^
2	1 sccm	Trigonal TiN	2.5 × 10^−4^
	1.5 sccm	Cubic TiN	2.5 × 10^−4^
	2 sccm	Cubic TiN	2.6 × 10^−4^
3	1 sccm	Cubic TiN	2.6 × 10^−4^
	2 sccm	Cubic TiN	2.6 × 10^−4^

**Table 3:** Stoichiometric composition, thickness, and surface roughness of TiN coating *.

Group	N_2_ Flow (sccm)	at. % N_2_	at. % Ti	Thickness (nm)	Ra (nm)	Rq (nm)
1	1	8.6 ± 0.2	91.4 ± 0.2	1630	16.0 ± 0.8	21 ± 1
	3	29.6 ± 0.1	70.4 ± 0.1	2027	7.6 ± 0.4	9.9 ± 0.5
	7	44.6 ± 0.2	55.4 ± 0.2	1724	4.5 ± 0.2	6.0 ± 0.3
	10	45.4 ± 0.2	54.6 ± 0.2	1550	2.2 ± 0.1	2.8 ± 0.1
	15	44.0 ± 0.1	56.0 ± 0.1	1317	2.4 ± 0.1	3.7 ± 0.2
2	1	2.6 ± 0.3	97.4 ± 0.3	2896	23 ± 1	29 ± 2
	1.5	16.4 ± 0.4	83.6 ± 0.4	1318	8.7 ± 0.4	11.1 ± 0.6
	2	40.8 ± 0.2	59.2 ± 0.2	1404	9.5 ± 0.5	12.5 ± 0.6
3	1	37.3 ± 0.2	62.7 ± 0.2	1080	5.4 ± 0.3	6.8 ± 0.3
	2	43.4 ± 0.8	56.6 ± 0.8	2200	17.5 ± 0.9	22 ± 1

* All obtained values for the studied areas of the coatings were averaged.

## Data Availability

The data presented in this study are available on request from the corresponding author. The data are not publicly available due to the ongoing character of the research.
